# MDA-9/Syntenin (SDCBP) modulates small GTPases RhoA and Cdc42 *via* transforming growth factor β1 to enhance epithelial-mesenchymal transition in breast cancer

**DOI:** 10.18632/oncotarget.13373

**Published:** 2016-11-15

**Authors:** Mitchell E. Menezes, Xue-Ning Shen, Swadesh K. Das, Luni Emdad, Devanand Sarkar, Paul B. Fisher

**Affiliations:** ^1^ Department of Human and Molecular Genetics, Virginia Commonwealth University, School of Medicine, Richmond, VA, USA; ^2^ VCU Institute of Molecular Medicine, Virginia Commonwealth University, School of Medicine, Richmond, VA, USA; ^3^ VCU Massey Cancer Center, Virginia Commonwealth University, School of Medicine, Richmond, VA, USA

**Keywords:** melanoma differentiation associated gene-9/syntenin, breast cancer, epithelial-mesenchymal transition, transforming growth factor beta 1

## Abstract

Epithelial-mesenchymal transition (EMT) is one of the decisive steps regulating cancer invasion and metastasis. However, the molecular mechanisms underlying this transition require further clarification. MDA-9/syntenin (SDCBP) expression is elevated in breast cancer patient samples as well as cultured breast cancer cells. Silencing expression of MDA-9 in mesenchymal metastatic breast cancer cells triggered a change in cell morphology in both 2D- and 3D-cultures to a more epithelial-like phenotype, along with changes in EMT markers, cytoskeletal rearrangement and decreased invasion. Conversely, over expressing MDA-9 in epithelial non-metastatic breast cancer cells instigated a change in morphology to a more mesenchymal phenotype with corresponding changes in EMT markers, cytoskeletal rearrangement and an increase in invasion. We also found that MDA-9 upregulated active levels of known modulators of EMT, the small GTPases RhoA and Cdc42, *via* TGFβ1. Reintroducing TGFβ1 in MDA-9 silenced cells restored active RhoA and cdc42 levels, modulated cytoskeletal rearrangement and increased invasion. We further determined that MDA-9 interacts with TGFβ1 *via* its PDZ1 domain. Finally, *in vivo* studies demonstrated that silencing the expression of MDA-9 resulted in decreased lung metastasis and TGFβ1 re-expression partially restored lung metastases. Our findings provide evidence for the relevance of MDA-9 in mediating EMT in breast cancer and support the potential of MDA-9 as a therapeutic target against metastatic disease.

## INTRODUCTION

The American Cancer Society estimates that in 2016, about 246,660 women will be diagnosed with breast cancer and approximately 40,450 women will die of the disease in the United States (American Cancer Society, Cancer Facts & Figures, 2016). Despite enhanced early detection, breast cancer is the second leading cause of cancer-related death among women in the United States. One of the reasons for this discrepancy is that treatment options are limited once primary tumors metastasize to distant areas in the body. Overall prognosis and patient survival are also adversely affected in metastatic disease. Consequently, it is imperative to identify relevant therapeutic targets that can inhibit metastasis of breast cancer. At the molecular level, epithelial-mesenchymal transition (EMT) enhances invasion and metastasis of cancer cells. EMT is a well conserved cellular process during which polarized, non-motile epithelial cells lose their polarized organization and cell-cell junctions and transition into motile mesenchymal cells [1, 2]. EMT is now widely accepted as a mechanism utilized by cancer cells to gain access to distant areas in the body [2-5]. Identifying distinctive molecules that regulate EMT and are “druggable” are thus critical to gain control of metastatic disease.

Melanoma differentiation associated gene-9 (MDA-9), also known as syntenin-1 (SDCBP; syndecan binding protein), is a member of the PDZ-domain containing family and is located on chromosome 8q12 [6]. Initially identified in our laboratory while screening for genes that were differentially expressed in human melanoma cells reprogrammed to terminally differentiate [7], MDA-9 has now been identified as a multifunctional protein involved in diverse physiological and pathological processes [8, 9]. MDA-9/syntenin plays an important role in several cellular functions including regulating cell-cell and cell-matrix adhesion, signal transduction from the cell surface to the interior through interaction with a number of proteins, intracellular and secreted lipid trafficking, and cell surface targeting [6, 9]. Recent studies also implicate MDA-9 as a key gene involved in cancer stem cell growth and survival [6, 9]. MDA-9 was also found to play a causative role in the progression of several different cancer types including melanoma [10-12], gastric cancer [13], bladder cancer [14], glioblastoma [15], small cell lung cancer [16], hepatoma [17] and head and neck cancers [18]. Recently, a study analyzing clinical patient samples found that MDA-9 expression was higher in patients with breast cancer and was associated with poor overall patient outcome [19]. Another study showed that MDA-9 regulates tumor cell growth in breast cancer [20]. However, the molecular mechanisms underlying the functional relevance and consequences of MDA-9 in breast cancer remains largely unexplored.

In the present study, we evaluated the role of MDA-9 in the invasive, metastatic and EMT abilities of breast cancer cells. We assessed the expression pattern of MDA-9 in breast cancer patient samples and breast cancer cell lines, and examined the impact of loss-of-function and gain-of-function of MDA-9 expression on metastatic and non-metastatic breast cancer cells. We further elucidated the molecular mechanism by which MDA-9 regulates EMT and metastasis in breast cancer. This is the first study that identifies the detailed molecular mechanism by which MDA-9 regulates EMT in breast cancer. Overall, our findings show that MDA-9 could provide a useful therapeutic and diagnostic target for breast cancer metastasis.

## RESULTS

### MDA-9 expression is elevated in human breast cancer

**Figure 1:**
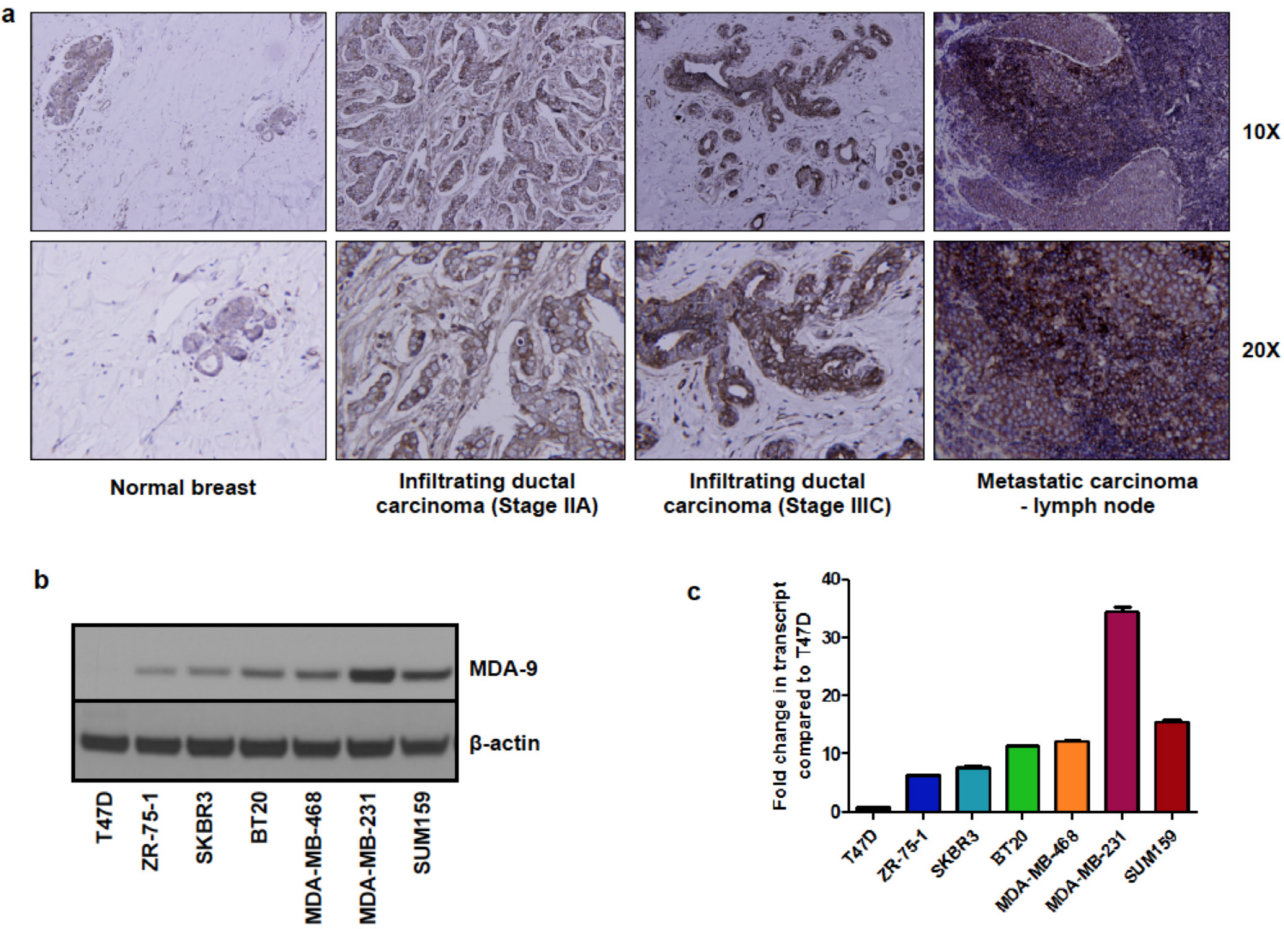
MDA-9 expression is elevated in breast cancer **a.** Immunohistochemistry of breast cancer patient samples showing overexpression of MDA-9 in breast cancer. **b.** MDA-9 protein expression is increased in invasive and metastatic cell lines. **c.** MDA-9 transcript expression is increased in invasive and metastatic breast cancer cell lines.

Metastatic breast cancer continues to pose a formidable problem for favorable patient outcome [21]. We recently demonstrated that MDA-9 plays a causative role in tumor progression and metastasis in melanoma [10, 11], urothelial [14], glioblastoma [15], and head and neck cancers [18]. To determine the role of MDA-9 in progression and metastasis of breast cancer, we assessed the expression of MDA-9 in patient samples and cell lines. A commercially available tissue microarray of breast cancer patient samples comprising adjacent normal breast tissue, breast tumor tissue and metastatic lesions was probed for MDA-9 expression by immunohistochemistry. Breast tumor tissue and metastatic lesions showed an increased expression of MDA-9 as compared to normal breast tissue (Figure 1a). This finding is in agreement with previous studies and was performed to verify previously published research to confirm that MDA-9/syntenin expression was elevated in breast cancer tissues and metastatic breast cancer cell lines [13, 19, 20]. A clinical study also found that elevated expression of MDA-9 correlated with increased metastasis and tumor recurrence in breast cancer patients [19]. This study showed that both overall survival and disease-free survival were reduced when MDA-9 expression was elevated [19]. We also assessed the DNA copy number of MDA-9 in the TCGA database to assess MDA-9 in a larger cohort of breast cancer patients (Supplementary Figure 1). MDA-9 DNA copy number was elevated in breast cancer patients as compared to normal controls in this larger cohort as well. Next, we assessed the expression of MDA-9 in a number of non-metastatic and metastatic human breast cancer cell lines and found that MDA-9 expression was elevated in metastatic cells at both the protein (Figure 1b) and transcript level (Figure 1c). Having validated that MDA-9 was indeed upregulated in breast cancer and was associated with increased metastatic incidence, we performed studies to understand the role of MDA-9 in the metastatic process in breast cancer.

### Modulating the expression of MDA-9 in breast cancer cells correlates with changes in invasive abilities and actin cytoskeletal rearrangement

**Figure 2:**
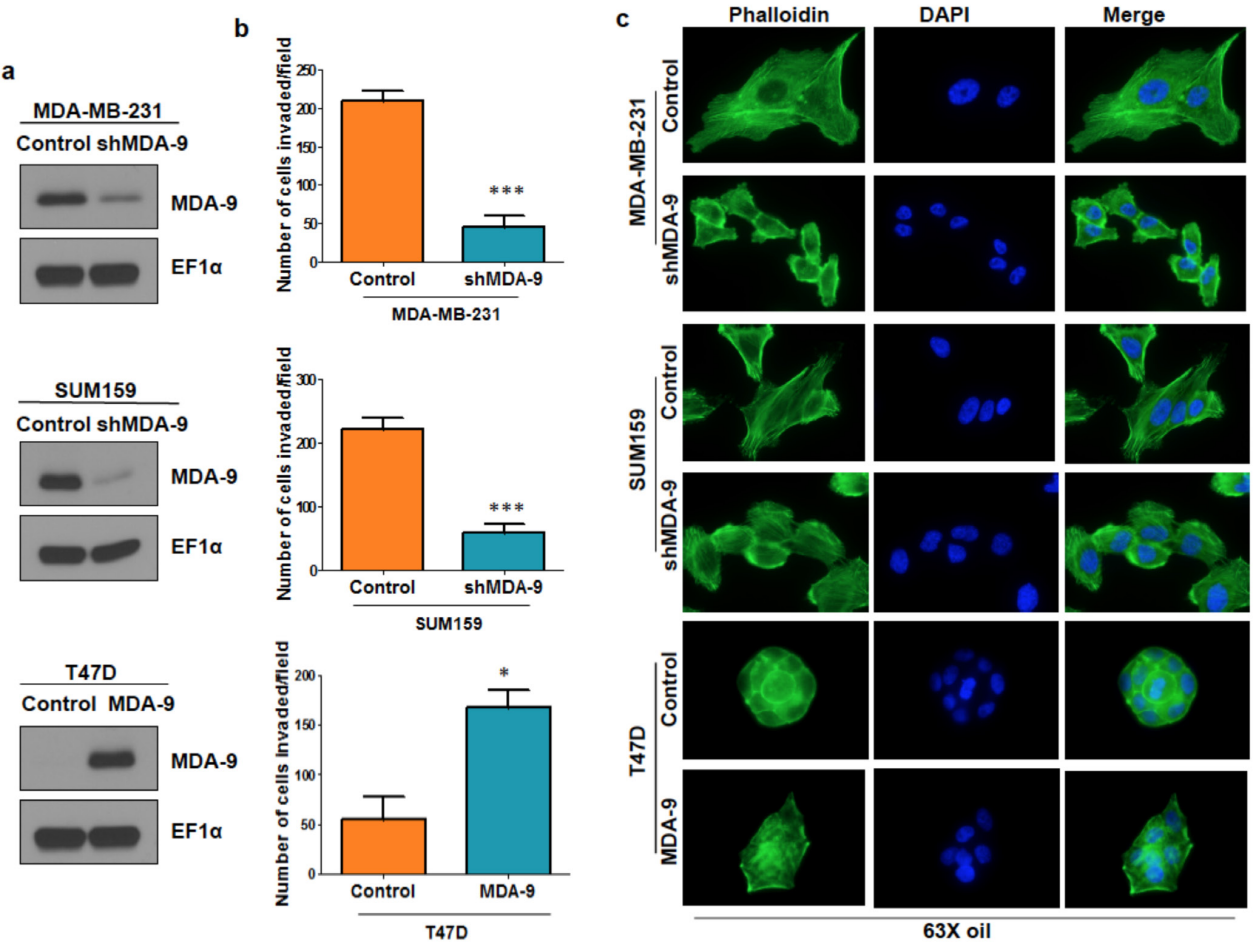
MDA-9 enhances invasion and cytoskeletal rearrangement **a.** Western blots showing efficient silencing of MDA-9 in MDA-MB-231 cells, efficient silencing of MDA-9 in SUM159 cells and efficient over expression of MDA-9 in T47D cells. **b.** Graphical representation of the invasion assay results in MDA-9 silenced MDA-MB-231 and SUM159 cells and MDA-9 overexpressing T47D cells. **c.** Representative images showing change in cytoskeletal reorganization following silencing of MDA-9 expression in MDA-MB-231 and SUM159 cells and overexpressing MDA-9 in T47D cells. *, *p* < 0.05; ***, *p*< 0.0001.

One of the hallmarks of cancer and particularly of metastasis, is the ability to invade into the surrounding basement membrane [4]. To investigate the importance of MDA-9 in metastatic breast cancer cells, we silenced MDA-9 expression in metastatic breast cancer cells, MDA-MB-231 and SUM159, using shRNA targeted to MDA-9 and non-targeted control (Figure 2a). Silencing the expression of MDA-9 caused a dramatic reduction in invasion in both MDA-MB-231 and SUM159 cells (Figure 2b). Next, we over expressed MDA-9 in non-metastatic breast cancer cells T47D using an adenovirus expressing MDA-9 and vector control. Overexpressing MDA-9 in T47D cells caused an increase in invasive abilities of these cells (Figure 2b). Reorganization of the cytoskeleton *via* polymerization and depolymerization of filamentous actin (F-actin) leads to changes in cell shape and assists in cell motility [1, 22]. We determined whether MDA-9 was able to regulate cytoskeletal rearrangement in breast cancer cells by staining for F-actin using phalloidin. Silencing the expression of MDA-9 in MDA-MB-231 and SUM159 cells caused a decrease in stress fibers, while overexpressing MDA-9 in T47D cells caused an increase in stress fiber formation (Figure 2c).

### Modulating the expression of MDA-9 in breast cancer cells correlates with changes in cell shape in 2D- and 3D-culture

**Figure 3:**
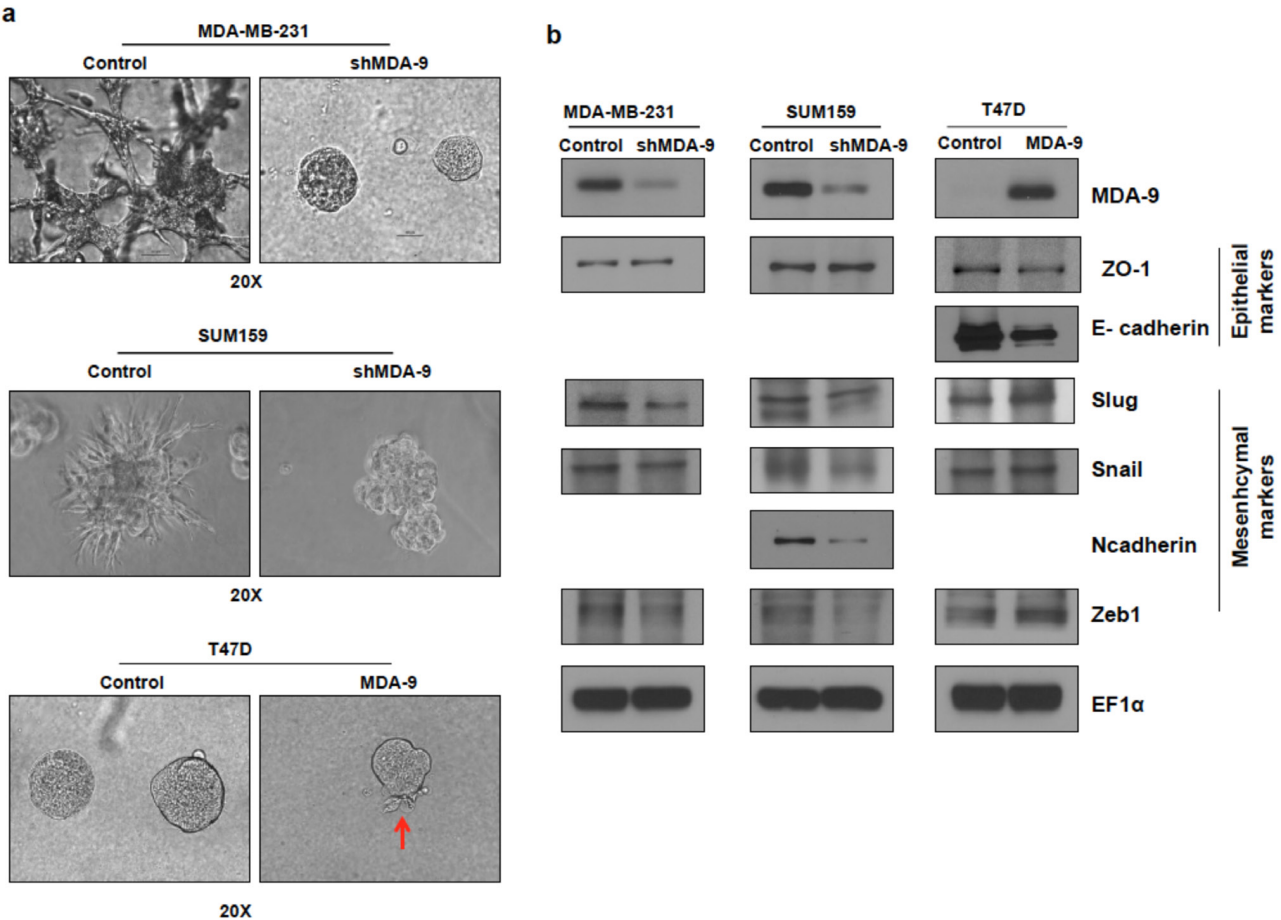
Silencing or overexpressing MDA-9 regulates EMT **a.** Representative images showing change in morphology in 3D culture following silencing MDA-9 expression in MDA-MB-231 and SUM159 cells and overexpressing MDA-9 in T47D cells. **b.** Western blots showing changes in key epithelial and mesenchymal markers following modulation of MDA-9 expression.

Modulating MDA-9 expression caused changes in cell shape in 2-dimensional (2D) culture conditions on plastic plates. Silencing the expression of MDA-9 in mesenchymal metastatic cells MDA-MB-231 and SUM159 caused the cells to appear epithelial-like (Supplementary Figure 2). Conversely, the epithelial T47D cells transitioned to a more mesenchymal phenotype following over expression of MDA-9 in 2D-culture. Next, the effects of modulating MDA-9 in cells grown in 3-dimensional (3D) culture conditions were assessed. Growing mammary epithelial cells in 3D-culture on a reconstituted basement membrane causes the cells to form spheroids that recapitulate several aspects of glandular architecture *in vivo* [23]. MDA-9 silenced cells formed compact spherical structures (spheroids) and lacked the invasive structures produced by the non-targeted control cells (Figure 3a). This indicates that MDA-9 silenced cells loose their ability to invade into the basement membrane and surrounding matrix. Conversely, when grown in 3D-culture conditions, unlike the compact spheroids observed in T47D control cells, T47D cells overexpressing MDA-9 produced projections, indicative of an increased ability to invade the basement membrane and surrounding matrix (Figure 3a).

### Modulating the expression of MDA-9 in breast cancer cells correlates with changes in EMT

Recent studies have identified EMT as the mechanism by which non-motile epithelial cancer cells progress towards more aggressive motile and invasive mesenchymal cells [1, 4]. Since MDA-9 enhanced invasive abilities and we observed a change in cell morphology both in 2D- and 3D-culture upon modulating the expression of MDA-9, which is indicative of EMT, we evaluated the role of MDA-9 in EMT and assessed the expression of several EMT markers in MDA-9 silenced metastatic breast cancer cells and MDA-9 overexpressing non-metastatic breast cancer cells (Figure 3b). Silencing the expression of MDA-9 caused a reduction in mesenchymal markers Slug, Snail and Zeb1 in both MDA-MB-231 and SUM159 cells. N-cadherin was also decreased in SUM159 cells. MDA-MB-231 cells are N-cadherin negative [24]. There was a slight increase in the epithelial marker ZO-1. Both SUM159 and MDA-MB-231 cells are E-cadherin negative [24]. Conversely, overexpressing MDA-9 in T47D cells resulted in a decrease in epithelial markers E-cadherin and ZO-1 and an increase in mesenchymal markers Slug, Snail and Zeb1. T47D cells are N-cadherin negative [25].

### MDA-9 modulates the small Rho GTPases RhoA and Cdc42 and enhances invasion and cytoskeletal rearrangement ***via*** TGFβ1

**Figure 4:**
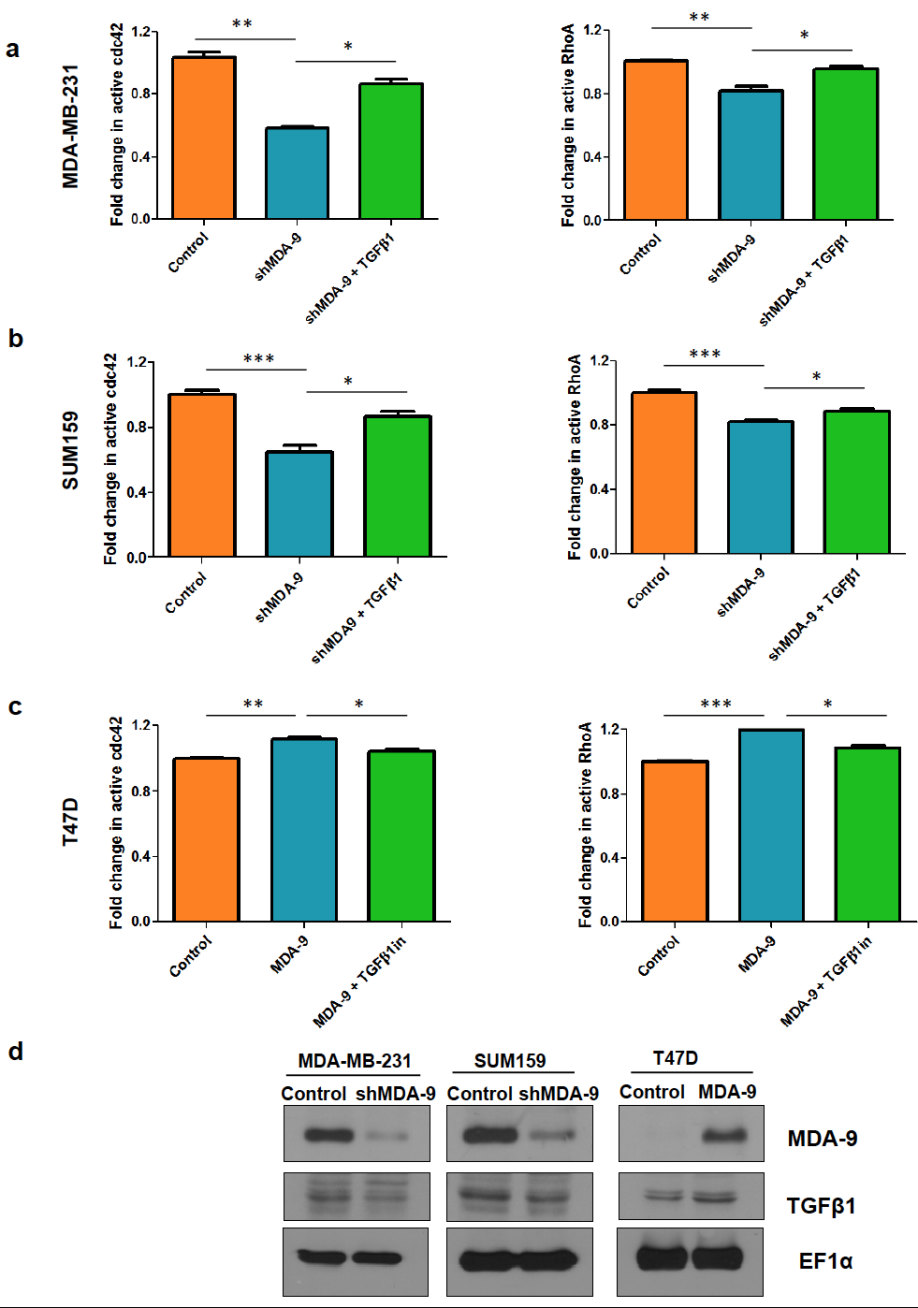
MDA-9 modulates small GTPases RhoA and Cdc42 via TGFβ1 Fold change in active cdc42 and RhoA levels in **a.** MDA-MB-231 cells and **b.** SUM159 cells after silencing MDA-9 expression and after re-introduction of TGFβ1. **c.** Fold change in active cdc42 and RhoA levels in T47D cells after overexpression of MDA-9 and after addition of TGFβ1 inhibitor **d.** Western blots showing changes in TGFβ1 expression following modulation of MDA-9 expression. *, *p* < 0.05; **, *p* < 0.01; ***, *p*< 0.0001.

Since the Rho family GTPases, RhoA and Cdc42, are known modulators of the actin cytoskeleton and play a vital role in EMT and metastasis in breast cancer [1, 26-28], we assessed the activity of these GTPases following MDA-9 modulation. We found that the active levels of RhoA and Cdc42 were downregulated when MDA-9 expression was silenced, while the active levels of RhoA and cdc42 were upregulated when MDA-9 was over expressed (Figure 4a, 4b, 4c - first two bars on each of the graphs).

**Figure 5:**
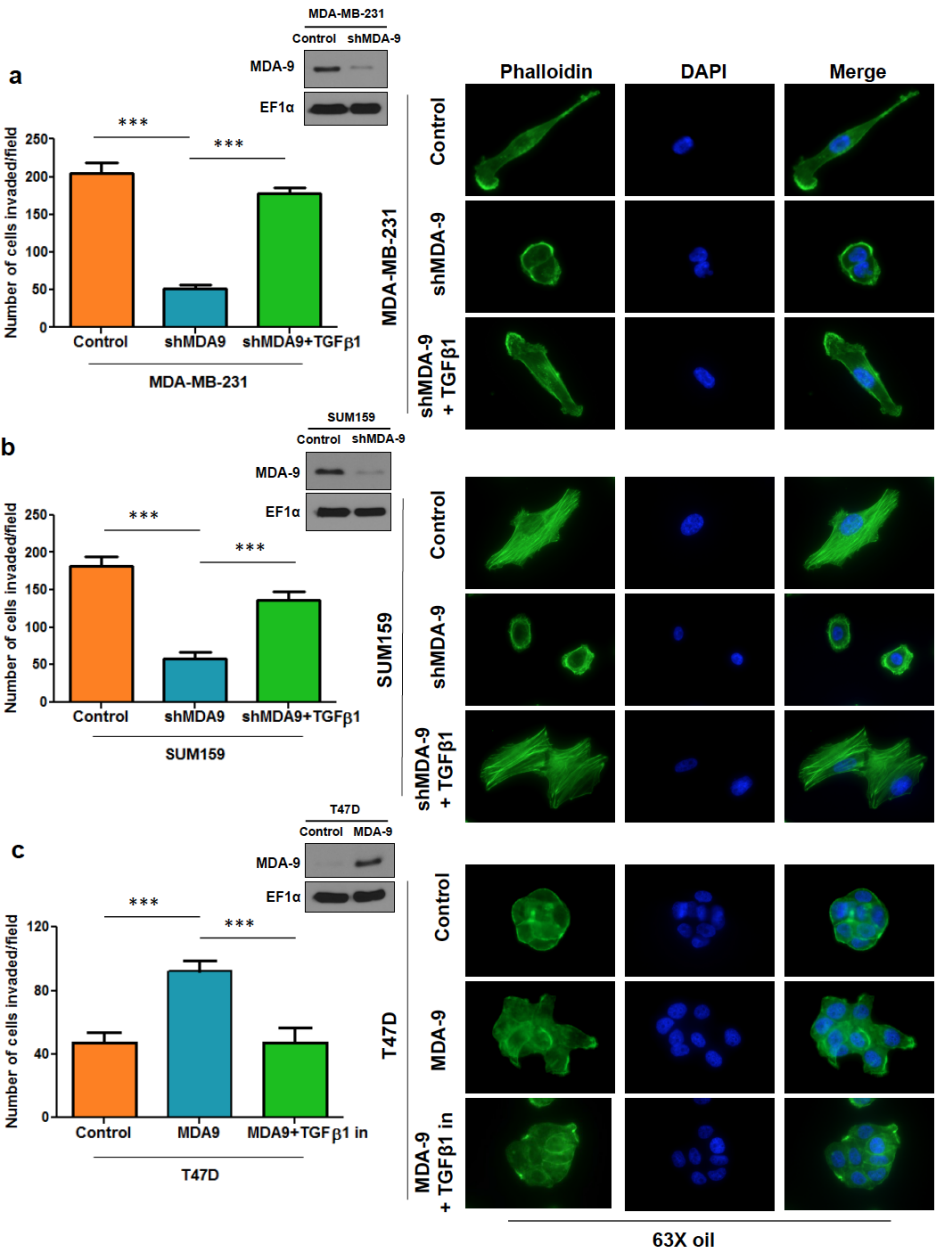
TGFβ1 modulation in MDA-9 modulated cells regulates invasion and cytoskeletal rearrangement Graphical representation of the invasion assay results and representative images showing cytoskeletal rearrangement upon re-introduction of TGFβ1 in MDA-9 silenced MDA-MB-231 **a.** and SUM159 **b.** cells. Inset: western blots showing efficient silencing of MDA-9. **c.** Graphical representation of the invasion assay results and representative images showing cytoskeletal rearrangement upon TGFβ1 inhibition in T47D cells overexpressing MDA-9. Inset: western blot showing efficient overexpression of MDA-9. ***, *p*< 0.0001.

One of the key modulators of RhoA and Cdc42 is TGFβ1 [29, 30]. To determine whether MDA-9/syntenin might regulate TGFβ1 to modulate RhoA and Cdc42 expression we assessed the expression levels of TGFβ1. We found that TGFβ1 levels were downregulated when MDA-9 expression was silenced while TGFβ1 levels were upregulated when MDA-9 was overexpressed (Figure 4d). To determine whether MDA-9 mediates its effects on RhoA and Cdc42 *via* TGFβ1, we re-introduced TGFβ1 in MDA-9 silenced cells and assessed active RhoA and Cdc42 levels (Figure 4a and 4b). Similarly, we inhibited TGFβ1 in T47D cells overexpressing MDA-9 and assessed the expression of RhoA and Cdc42 (Figure 4c). To further validate these findings in relation to the role of MDA-9 in invasion, we found that re-introducing TGFβ1 in MDA-9 silenced cells restored the invasive abilities of MDA-MB-231 and SUM159 cells and caused cytoskeletal rearrangement (Figure 5a and 5b). Conversely, inhibiting TGFβ1 expression in MDA-9 overexpressing cells resulted in a decrease in invasive abilities and cytoskeletal rearrangement (Figure 5c).

### PDZ1 domain of MDA-9 interacts with TGFβ1

Next, we endeavored to determine how MDA-9 regulates TGFβ1. To assess whether MDA-9 regulates the transcription of TGFβ1, we performed luciferase reporter assays using TGFβ1 promoter luciferase constructs, but did not find an increase in luciferase reporter activity (data not shown). This indicates that MDA-9 did not regulate TGFβ1 at the transcriptional level. We searched the Oncomine database to determine any association between MDA-9 and TGFβ1 in breast cancer patient populations. Interestingly, we found a dataset that showed that MDA-9 and TGFβ1 were co-expressed in a set of breast cancer patients (Supplementary Figure 3). We also found that Slug (SNAI2), a well-known EMT-inducing transcription factor, was also co-expressed with MDA-9 and TGFβ1, further supporting our overall hypothesis. Next we determined the DNA copy number of TGFβ1 in the same breast cancer patient cohort that was assessed for MDA-9 DNA copy number in Supplementary Figure 1 (Supplementary Figure 4). We observed an association between the DNA copy numbers of MDA-9 and TGFβ1 in the breast cancer patient samples.

**Figure 6:**
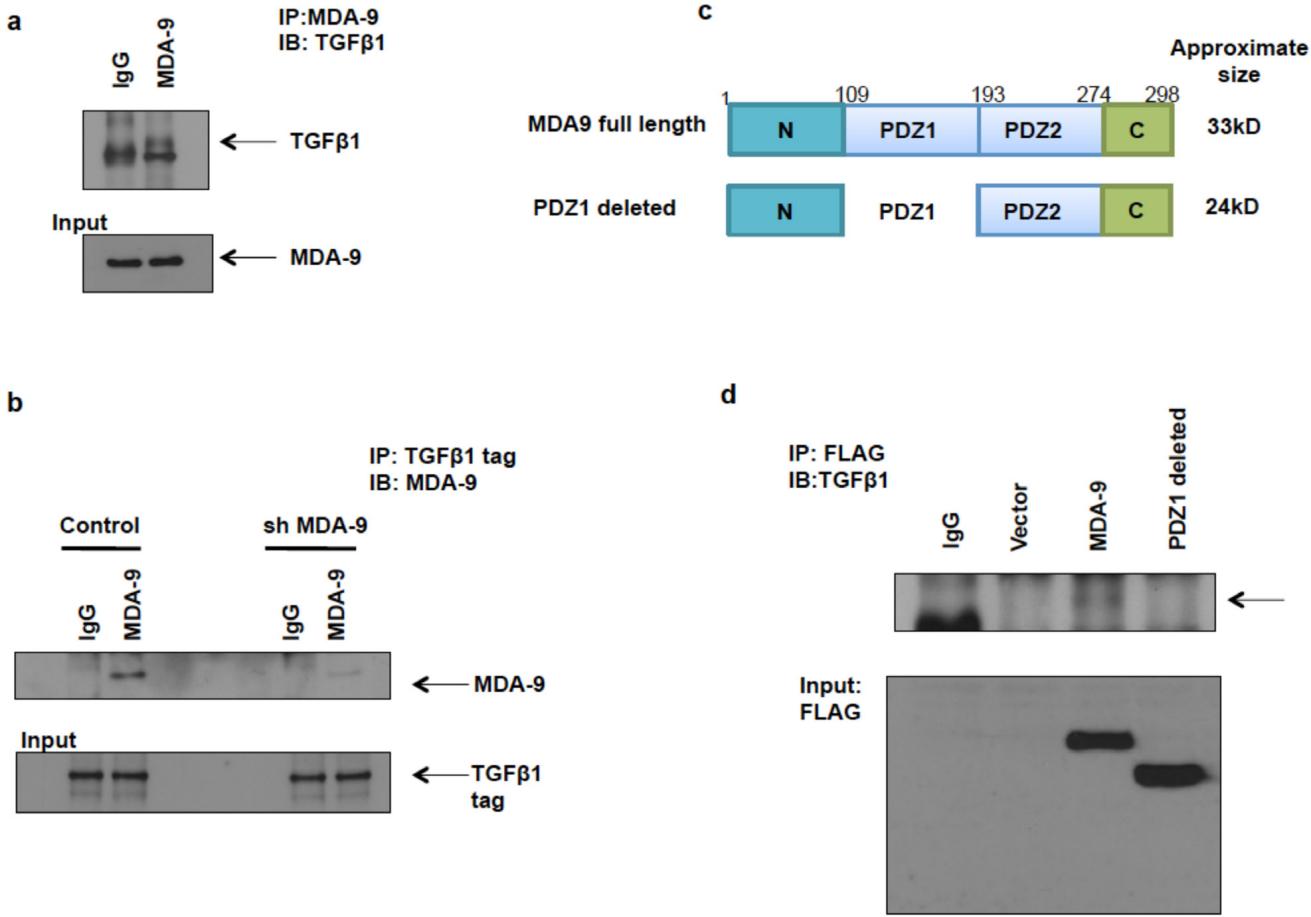
MDA-9 interacts with TGFβ1 **a.** Immunoprecipitation with MDA-9 antibody and immunoblotting using TGFβ1 antibody showed that MDA-9 interacts with TGFβ1. **b.** Immunoprecipitation with TGFβ1 tag antibody and immunoblotting with MDA-9 antibody showed that TGFβ1 interacts with MDA-9 and this interaction was decreased when MDA-9 expression was silenced. **c.** Schematic representation of full length MDA-9 constructs and PDZ1 deleted constructs (with FLAG tag). **d.** Immunoprecipitation with FLAG antibody and immunoblotting with TGFβ1 antibody shows that PDZ1 domain of MDA-9 interacts with TGFβ1.

To validate these findings, we performed coimmunoprecipitation assays and determined that MDA-9 physically interacted with TGFβ1. First, we performed immunoprecipitation in SUM159 cells using the MDA-9 antibody and immunoblotted with the TGFβ1 antibody and found that MDA-9 interacts with TGFβ1 (Figure 6a). Next we performed immunoprecipitation in SUM159 control cells overexpressing TGFβ1 and SUM159 cells silenced for MDA-9 expression and overexpressing TGFβ1 in order to easily pull down TGFβ1 using the TGFβ1 tag and immunoblotted using the MDA-9 antibody and found that MDA-9 interacted with TGFβ1 (Figure 6b). The MDA-9 - TGFβ1 interaction was decreased in MDA-9 silenced cells, further validating our observations. Next, we determined the region of MDA-9 that was involved in the interaction with TGFβ1. Figure 6c shows the full length MDA-9 construct and a PDZ1 deleted version of MDA-9. Deletion of the PDZ1 domain disrupts the interaction between MDA-9 and TGFβ1 (Figure 6d). This indicates that the PDZ1 domain is essential for its interaction with TGFβ1.

### Silencing the expression of MDA-9 in metastatic breast cancer cells causes a decrease in lung metastases ***in vivo***, which could be partially reversed by TGFβ1 restoration

**Figure 7:**
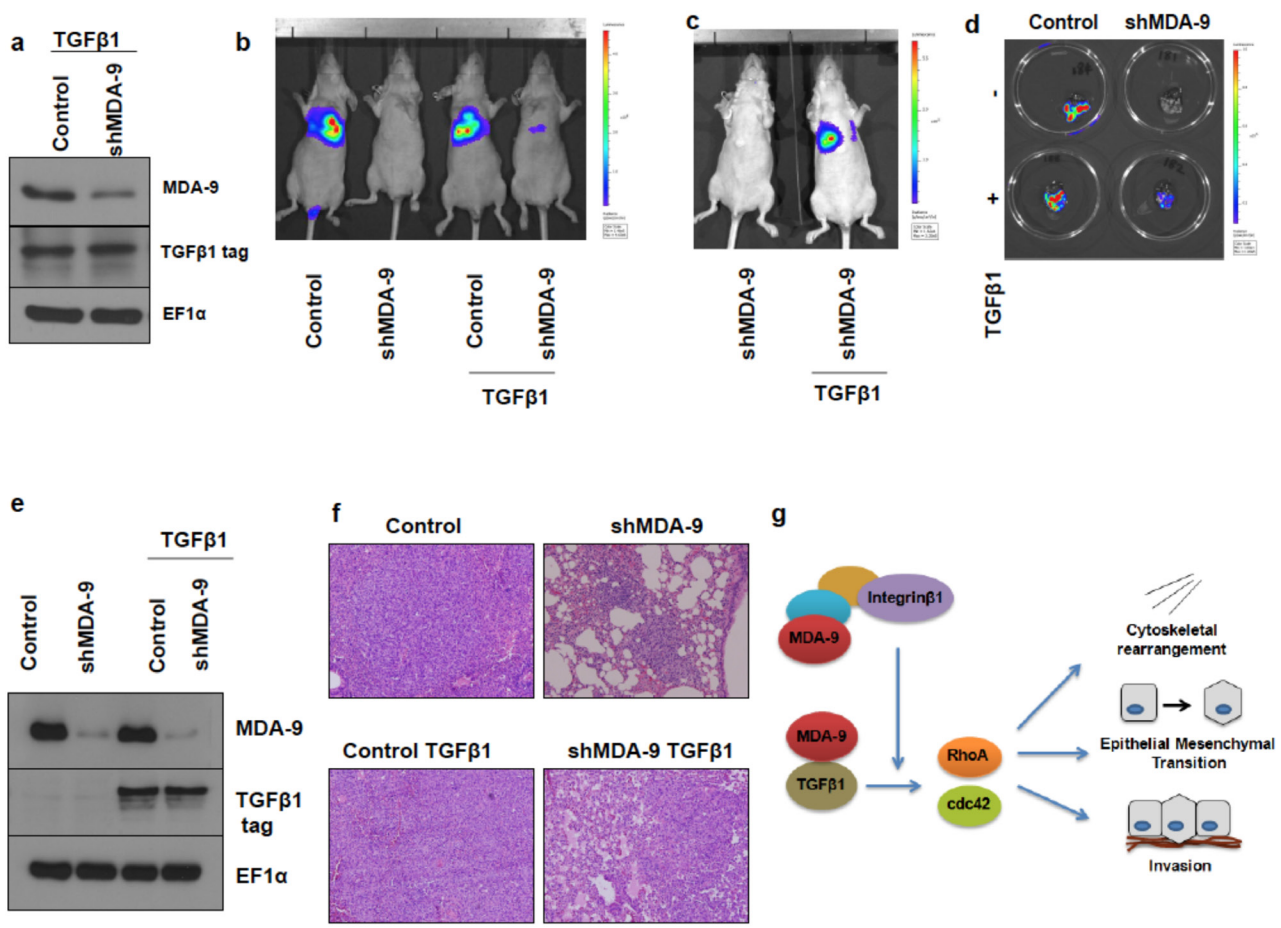
Silencing MDA-9 causes a reduction in lung metastasis, which can be rescued by restoration of TGF**β**1 expression **a.** Western blot images showing stable expression of TGFβ1 in MDA-MB-231 control TGFβ1 luciferase cells and MDA-MB-231 shMDA-9 TGFβ1 luciferase cells. **b.** Bioluminescence imaging showing reduction of lung metastasis in mice injected with MDA-MB-231 shMDA-9 luciferase cells and rescue following re-expression of TGFβ1. **c.** Bioluminescence imaging showing rescue of lung metastasis in mice injected with MDA-MB-231 shMDA-9 TGFβ1 luciferase cells compared to mice injected with MDA-MB-231 shMDA-9 luciferase cells. **d.** Bioluminescent images of the lungs showing metastasis. **e.** Western blot images of cells isolated from the respective lungs and probed for MDA-9 and TGFβ1 tag expression. **f.** H&E images of the lung sections showing presence of lung metastases. MDA-MB-231 control luciferase and MDA-MB-231 control TGFβ1 luciferase cells efficiently colonized the entire lungs. MDA-MB-231 shMDA-9 luciferase cells formed a few small lung metastases while MDA-MB-231 shMDA-9 TGFβ1 luciferase cells showed partial restoration of metastatic capabilities and formed multiple larger lung metastatic lesions. **g.** Schematic representation of the signaling mechanism mediated by MDA-9 to regulate cytoskeletal rearrangement, EMT and invasion. MDA-9 has previously been shown to regulate the formation of various integrin β1 signaling complexes. Integrin β1, in turn, functions to enhance TGFβ1-mediated non-canonical signaling and EMT and blocking integrin β1 function inhibited TGFβ-mediated non-canonical signaling and EMT. In breast cancer cells, MDA-9 interacts with TGFβ1 and regulates the small GTPases RhoA and cdc42 *via* TGFβ1. Further, MDA-9 regulates EMT and invasion via TGFβ1.

To further validate our findings that MDA-9 was able to enhance the metastatic potential of breast cancer, we performed *in vivo* lung metastasis studies. We developed MDA-MB-231 control, MDA-MB-231 shMDA-9, MDA-MB-231 control TGFβ1 and MDA-MB-231 shMDA-9 TGFβ1 cells stably expressing luciferase (Figure 7a). By incorporating luciferase into these MDA-MB-231 cell lines we were able to monitor development of lung metastasis *via* bioluminescent imaging (BLI). MDA-MB-231 control luciferase cells were able to colonize the lungs and establish metastases following introduction into athymic mice intravenously *via* the tail vein (Figure 7b). MDA-MB-231 shMDA-9 luciferase cells however showed a dramatic reduction in the ability to colonize and establish metastases in the lungs. As would be expected, MDA-MB-231 control TGFβ1 luciferase cells were also able to colonize the lungs and establish lung metastases. Importantly, re-expressing TGFβ1 in MDA-MB-231 cells silenced for the expression of MDA-9 caused a partial restoration of lung metastases (Figure 7c and 7d). Further, tumor cells were harvested from the lung metastases that developed in the athymic mice and re-grown in culture. These cells were evaluated for expression of MDA-9 and TGFβ1. MDA-MB-231 cells that were silenced for MDA-9 expression continued to show very low MDA-9 expression and TGFβ1expressing cells showed TGFβ1 expression (Figure 7e). Figure 7f shows H&E staining of the tumor sections showing presence of lung metastases. The entire lungs were colonized by both MDA-MB-231 control luciferase cells and MDA-MB-231 control TGFβ1 luciferase cells. However only a few small lung metastases were observed in the H&E sections of the lungs injected with MDA-MB-231 shMDA-9 cells. Re-expressing TGFβ1 in MDA-MB-231 cells allowed these cells to form larger lung metastatic lesions as observed in the H&E section.

## DISCUSSION

While widespread awareness regarding breast cancer has facilitated early detection and improved overall patient outcomes, patient prognosis is adversely affected when breast cancer metastasizes to distant areas in the body. Hence, identifying novel therapeutic targets that inhibit EMT and metastasis are key elements in effectively targeting metastatic breast cancer. We report here that MDA-9 is upregulated in breast cancer as well as metastases and plays a key role in EMT induction in breast cancer cells. We further provide detailed mechanistic insights into the signaling mediated by MDA-9 to enhance EMT.

MDA-9 is a scaffold protein with multiple diverse roles in tumorigenesis, particularly, in tumor invasion and metastasis. MDA-9 was found to be over expressed in a number of human cancers including melanoma [10-12], gastric cancer [13], bladder cancer [14], glioblastoma [15], small cell lung cancer [16], hepatoma [17], head and neck cancers [18] and breast cancer [19]. Due to its seminal role in several cancers, researchers have focused on dissecting the signaling mechanisms regulated by MDA-9. In the various cancer types evaluated, MDA-9 orchestrates cancer attributes *via* its interaction with key binding partners including several oncogenic proteins [9, 31]. In melanoma cells, MDA-9 colocalizes with focal adhesion kinase (FAK), a key component of integrin-mediated signaling pathways, and increased phosphorylation of FAK, c-Jun-NH2-kinase (JNK) and p38 MAPK [10]. MDA-9 was also shown to interact with c-Src, which enhanced FAK/c-Src complex formation and activated c-Src [11]. Studies using glioblastoma cells also showed that MDA-9 increased the activation of c-Src, p38 MAPK and nuclear factor kappa B (NF-κB), which enhanced expression of matrix metalloproteinase 2 (MMP2) and the secretion of interleukin-8 (IL-8) [15]. In urothelial cancer cells, MDA-9 interacts with EGFR and enhanced the expression of EGFR, AKT, phosphoinositide 3-kinase (PI3K) and c-Src [14]. In head and neck squamous cell carcinoma, MDA-9 colocalized with VEGFR1 and regulated the expression of SPRR1B and VEGFR1. Growth regulatory molecules including Cyclin D1, CDK4, STAT3, PI3K and CTNNB1 were also modulated by MDA-9 [18].

The detailed mechanism by which MDA-9 regulates invasion and metastasis of breast cancer remains largely unknown. The findings from our study provide insights into the signaling mechanisms regulated by MDA-9 in breast cancer. We show that MDA-9 expression correlated with invasiveness and metastatic capabilities of breast cancer cells. Loss-of-function and gain-of-function studies confirmed the relevance of MDA-9 in EMT, invasion and cytoskeletal rearrangement and helped elucidate the molecular mechanisms of action of MDA-9. We demonstrate that MDA-9 regulates the small GTPases RhoA and Cdc42 *via* TGFβ1. Researchers have shown that treating prostate cancer cells with TGFβ1 induced rapid formation of lamellipodia and cytoskeletal rearrangements [32]. Importantly, this response to TGFβ1 was independent of canonical Smad signaling and required the activity of small Rho GTPases Cdc42 and RhoA [32]. This study, albeit in a different cancer indication, supports our findings and suggest that MDA-9 could act upstream of TGFβ1 to mediate cytoskeletal rearrangements.

We further show that MDA-9 could interact with TGFβ1. We also observed that MDA-9 and TGFβ1 were co-expressed in breast cancer patient samples in the TCGA database using Oncomine (Supplementary Figure 3). Re-introducing TGFβ1 in MDA-9-silenced cells caused a partial restoration of invasive abilities. Similarly, restoring TGFβ1 expression in MDA-9-silenced cells caused a partial restoration of lung metastases *in vivo*. Interestingly, a study in A549 lung carcinoma cells showed that MDA-9 might also prevent caveolin-1-mediated internalization of TGFβR1 leading to enhanced canonical TGFβ1 signaling [33]. These findings provide evidence for another layer of regulation of TGFβ1 signaling by MDA-9 supporting our overall hypothesis. MDA-9 is a known scaffold protein and the MDA-9-TGFβ1 interaction might also serve to stabilize the TGFβ1 protein. Further investigation into the mechanism by which MDA-9 regulates TGFβ1 is ongoing. We also observed that MDA-9 and Slug (SNAI2) were co-expressed in breast cancer patient samples in the TCGA database using Oncomine (Supplementary Figure 3). A very recent study in lung adenocarcinoma showed that MDA-9 interacts with Slug and regulates invasion and metastasis, further validating our findings [34]. Further, studies have identified a link between triple negative breast tumors and the occurrence of EMT [35, 36]. From the observations in Figure 1b and 1c and the understanding we have gained regarding the role of MDA-9 in breast cancer in this paper, one can speculate that there might be an association between the expression of MDA-9 and triple negative breast cancer. Further experimentation is required to confirm this hypothesis.

Interestingly, integrin β1 has been shown to play a role in aiding TGFβ1-mediated non-canonical signaling including downstream RhoA and Cdc42 signaling [37]. Additionally, MDA-9 was shown to play a key role in stabilizing integrin β1 signaling complexes [38]. Hence, we wondered whether integrin β1 might also be involved in this MDA-9-TGFβ1 signaling axis. The role of the extracellular matrix, comprised of integrins, cannot be ignored when trying to understand tumor attributes such as invasion [39]. In fact, integrins are key players that enhance breast cancer invasion and metastasis [40, 41] and integrin β1 plays a key role in metastatic progression of breast cancer [42-44]. Hence we added an integrin β1 blocking antibody to SUM159 cells and assessed cytoskeletal rearrangement (Supplementary Figure 5a). Addition of integrin β1 blocking antibody caused a change in cytoskeletal organization. Next, we added an integrin β1 blocking antibody to T47D cells overexpressing MDA-9 and observed a change in cytoskeletal organization (Supplementary Figure 5b). Our observations and previous studies indicate that integrin β1 might also be involved in MDA-9 mediated regulation of the small GTPases RhoA and Cdc42. This finding is consistent with published reports that show that MDA-9 plays a key role in the assembly of integrin β1 signaling complexes and that silencing the expression of MDA-9 impaired assembly/formation of several integrin β1 signaling complexes [38, 45]. Additionally, silencing the expression MDA-9 also resulted in inhibition of active integrin β1 expression (and downstream phosphorylation of ERK1/2) in breast cancer cells [19]. Thus, the overall signaling mechanism mediated by MDA-9 in breast cancer is illustrated in Figure 7g.

In summary, our study has identified a novel role of MDA-9 in mediating EMT and enhancing invasive abilities in breast cancer cells. Additionally, our findings provide preclinical evidence that MDA-9 might be an effective therapeutic target against breast cancer including metastatic breast cancer.

## MATERIALS AND METHODS

### Cell lines and cell culture

Human breast cancer cells T47D, ZR-75-1, SKBR3, BT-20, MDA-MB-468 and MDA-MB-231 were purchased from the American Type Culture Collection (ATCC) (Manassas, VA) and cultured as recommended by ATCC. T47D, ZR-75-1 and SKBR3 are epithelial cells with low invasive and metastatic ability. BT-20 and MDA-MB-468 are moderately invasive and metastatic. MDA-MB-231 is a highly invasive and metastatic triple negative mesenchymal breast cancer cell line [46, 47]. Human breast cancer cells SUM159PT (labeled SUM159 throughout) were purchased from Asterand, Inc. (Detroit, MI). These cells were cultured in F-12 media supplemented with 5% fetal bovine serum, 10mM HEPES buffer, 5μg/ml insulin, 1μg/ml hydrocortisone and 1% Penicillin/Streptomycin. SUM159 cells are triple negative with strong abilities to invade and metastasize [48]. ATCC authenticates these cell lines using short tandem repeat analysis. All the cell lines were expanded and frozen immediately after receipt. The cumulative culture length of the cells was less than 6 months after recovery. Early passage cells were used for all experiments. All the cell lines were frequently tested for mycoplasma contamination using a mycoplasma detection kit from Sigma. All of the cells were maintained at 37°C with 5% CO_2_ in a humidified atmosphere.

### Breast cancer tissue microarray

Tissue microarray comprised of breast cancer samples along with metastatic and normal counterpart tissue samples was purchased from Imgenex (San Diego, CA). Immunohistochemistry was performed according to standard protocols. Briefly, tumor sections were deparaffinized at 60°C for 1 hour, followed by rehydration, and antigen retrieval using citrate buffer and heating. Avidin and biotin blocking kits and Vectastain ABC complex kits were obtained from Vector Laboratories (Burlingame, CA). IHC-grade Prestige MDA-9 antibody was purchased from Sigma (St. Louis, MO). Secondary antibodies were obtained from Jackson Immunoresearch (West Grove, PA). The slides were counter-stained using hematoxylin. Following staining, slides were dehydrated and mounted using Vectashield mounting media (Vector Laboratories).

### Preparation of whole-cell lysates and western blotting analysis

Cells were washed twice with ice-cold PBS and lysed in 1X Cell Lysis buffer (Cell Signaling Technology, Danvers, MA) with protease and phosphatase inhibitors. The lysates were kept on ice for 1 hour and centrifuged at 10,000 rpm for 30 minutes at 4°C. Protein concentration was measured using BioRad protein assay reagent (Hercules, CA). Lysates corresponding to equal amounts of protein were subjected to SDS-PAGE and transferred on to PVDF membrane (0.2 μm). The membranes were blocked with 5% non-fat dried milk or bovine serum albumin (BSA) in TBST (Tris-buffered saline with 0.1% Tween 20) and incubated with primary antibodies overnight at 4°C. The membranes were then washed three times with TBST, incubated with respective secondary antibodies for 1 hour at room temperature, washed three times with TBST and then developed using ECL reagent (GE Healthcare, UK). MDA-9/syntenin antibody was from Abnova (Taiwan), β-actin was from Sigma-Aldrich (St. Louis, MO), EMT marker antibodies were from Cell Signaling Technologies and TGFβ1 tag antibody was from Origene (Rockville, MD). EF1α was used as a loading control and was obtained from EMD Millipore (Billerica, MA).

### RNA extraction and qRT-PCR (quantitative real-time PCR)

Cells were washed with PBS and then harvested using Qiazol. RNAeasy kit (Qiagen, Germany) was used for RNA extraction according to the manufacturer's protocol. RNA was converted to cDNA using the cDNA synthesis kit (Applied Biosystems, Foster City, CA). The PCR primers and probes were purchased from Applied Biosystems. qRT-PCR was performed using the Applied Biosystems machine. The gene expression Δ*C*_T_ values of mRNAs from each sample were calculated by normalizing with GAPDH and relative quantification values were plotted using GraphPad Prism^®^.

### Viruses, plasmids and reagents

MDA-9 was silenced using Ad.5/3 shMDA-9 and overexpressed using Ad.5/3 MDA-9. The construction of these viruses has been described in detail previously [49]. For stably silencing the expression of MDA-9, lentiviruses targeting MDA-9 were purchased from Sigma (St. Louis, MO). FLAG-tagged full length and PDZ-1 deleted constructs were obtained from TransOMIC (Huntsville, AL). TGFβ1 construct was obtained from Origene. Human TGFβ1 protein was obtained from BioLegend (San Diego, CA) and the TGFβ1 inhibitor (A83-01) was obtained from (Stemgent, San Diego, CA).

### Invasion assay

Invasion assays were performed using 24-well BioCoat Matrigel™ invasion chambers (BD Biosciences, San Jose, CA) in triplicates according to the manufacturer's instructions. Briefly, the chambers were equilibrated to room temperature and then rehydrated using warm serum-free cell culture media for 2 hours at 37°C. Cells were seeded in serum-free media in the upper chamber and serum-containing media was used as an attractant in the lower chamber. Cells were allowed to invade through the Matrigel™ overnight. The inserts were fixed with methanol, non-invaded cells were wiped off using a cotton swab and the inserts along with the invaded cells were stained with the Diff-Quick staining kit. Cells that invaded were enumerated using at least 8 fields per insert. Data is presented as mean ± S.E.M.

### Phalloidin staining

Cells were seeded in 4-well chambered glass slides and allowed to attach overnight. The next day, the cells were fixed with 4% paraformaldehyde for 1 hour, and permeabilized with 0.01% Triton-X and 1% sodium citrate for 3 minutes on ice. The cells were blocked with 1% bovine serum albumin for 30 minutes. The actin cytoskeleton was stained using Alexa Fluor 488 Phalloidin (Life Technologies, Carlsbad, CA) for 30 minutes. The wells were detached from the glass slide and the slide was mounted using mounting media containing DAPI (to label cell nuclei) (Vector Laboratories).

### 3D (three-dimensional) culture

Glass slides (eight-well chambered; Nunc, Rochester, NY) were coated with 3D Culture Matrix™ Basement Membrane Extract Reduced Growth Factor (Phenol Red-free) (Trevigen,). A total of 5000 cells/well were seeded into the wells in complete medium containing 2% 3D Matrix and the media was replenished every 4 days. The slides were incubated at 37°C, humidified with 5% CO_2_ atmosphere [23, 50].

### GTPase activity assay

The RhoA G-LISA and Cdc42 G-LISA activation assay biochem kits were obtained from Cytoskeleton Inc (Denver, CO) and GTPase activity was measured according to the manufacturer's instructions. Briefly, cells were cultured in serum-free media for 48 hours and lysates were prepared using the appropriate G-LISA buffers. The required number of G-LISA wells were rehydrated with ice-cold water, and then lysates, lysis buffer (negative control) and RhoA/Cdc42 control protein (positive control) were added to the wells. The plate was incubated at 4°C, followed by incubation with antigen presenting buffer, primary antibody, secondary antibody and then the HRP detection reagents. After adding the HRP stop buffer, absorbance was measured at 490nm.

### Immunoprecipitation

Immunoprecipitation was performed using the immunoprecipitation kit with Dynabeads^®^ Protein G (Life Technologies) according to the manufacturer's instructions. Briefly, MDA-9, TGFβ1, FLAG tag or IgG control antibody was incubated with Dynabeads^®^ and allowed to bind. Next, protein lysates were incubated with the antibody bound Dynabeads^®^. Finally, the antibody-antigen complexes were eluted from the Dynabeads^®^, run on an SDS-PAGE gel and probed using the appropriate antibody.

### *In vivo* metastasis study

MDA-MB-231 control cells were stably transfected with the luciferase expression plasmid pGL4.50 (Promega, Madison, WI) specifically engineered to aid *in vivo* imaging. MDA-MB-231 control cells stably expressing luciferase were selected using hygromycin. MDA-9 stably silenced cells were similarly stably transfected with the luciferase expressing plasmid. Both MDA-MB-231 control luciferase and MDA-MB-231 shMDA-9 luciferase cells were stably transfected with TGFβ1. 6-week old female athymic mice (Charles River Laboratories, Wilmington, MA) were injected with 1.5x10^6^ cells of the above four cell lines through the tail-vein. Five female athymic mice were injected per cell line. The mice were monitored and luciferase expression was assessed by bioluminescent imaging. The mice were sacrificed after 6 weeks. A section of the lung was collected in DMEM/F-12 media supplemented with Penicillin/Streptomycin. Tumor cells were harvested from the lung metastasis by digesting the lung tissue using trypsin-EDTA. Tumor cells were collected and cultured as per normal procedures. Another section of the lungs was fixed in paraffin, sectioned and H&E stained. Animals were maintained under the guidelines of the National Institute of Health and under evaluation and approval of the Institutional Animal Care and Use Committee (Virginia Commonwealth University). Food and water were provided *ad libitum*.

### Statistical analysis

Results are presented as the mean ± S.E.M. for at least three individual experiments. Statistical analyses were performed using GraphPad Prism 5. Student's t-test was applied based on the statistical mandates or suggestions of each analysis. p<0.05 was considered statistically significant.

## SUPPLEMENTARY MATERIAL



## References

[1] Lamouille S, Xu J, Derynck R (2014). Molecular mechanisms of epithelial-mesenchymal transition. Nature reviews Molecular cell biology.

[2] Kalluri R, Weinberg RA (2009). The basics of epithelial-mesenchymal transition. J Clin Invest.

[3] Yang J, Weinberg RA (2008). Epithelial-mesenchymal transition: at the crossroads of development and tumor metastasis. Developmental cell.

[4] Hanahan D, Weinberg RA (2011). Hallmarks of cancer: the next generation. Cell.

[5] Ye X, Weinberg RA (2015). Epithelial-Mesenchymal Plasticity: A Central Regulator of Cancer Progression. Trends in cell biology.

[6] Sarkar D, Boukerche H, Su ZZ, Fisher PB (2008). mda-9/Syntenin: more than just a simple adapter protein when it comes to cancer metastasis. Cancer research.

[7] Lin JJ, Jiang H, Fisher PB (1998). Melanoma differentiation associated gene-9, mda-9, is a human gamma interferon responsive gene. Gene.

[8] Kegelman TP, Das SK, Emdad L, Hu B, Menezes ME, Bhoopathi P, Wang XY, Pellecchia M, Sarkar D, Fisher PB (2014). Targeting tumor invasion: the roles of MDA-9/Syntenin. Expert Opin Ther Targets.

[9] Das SK, Bhutia SK, Kegelman TP, Peachy L, Oyesanya RA, Dasgupta S, Sokhi UK, Azab B, Dash R, Quinn BA, Kim K, Barral PM, Su ZZ, Boukerche H, Sarkar D, Fisher PB (2012). MDA-9/syntenin: a positive gatekeeper of melanoma metastasis. Front Biosci (Landmark Ed).

[10] Boukerche H, Su ZZ, Emdad L, Baril P, Balme B, Thomas L, Randolph A, Valerie K, Sarkar D, Fisher PB (2005). mda-9/Syntenin: a positive regulator of melanoma metastasis. Cancer research.

[11] Boukerche H, Su ZZ, Prevot C, Sarkar D, Fisher PB (2008). mda-9/Syntenin promotes metastasis in human melanoma cells by activating c-Src. Proceedings of the National Academy of Sciences of the United States of America.

[12] Gangemi R, Mirisola V, Barisione G, Fabbi M, Brizzolara A, Lanza F, Mosci C, Salvi S, Gualco M, Truini M, Angelini G, Boccardo S, Cilli M, Airoldi I, Queirolo P, Jager MJ (2012). Mda-9/syntenin is expressed in uveal melanoma and correlates with metastatic progression. PLoS One.

[13] Koo TH, Lee JJ, Kim EM, Kim KW, Kim HD, Lee JH (2002). Syntenin is overexpressed and promotes cell migration in metastatic human breast and gastric cancer cell lines. Oncogene.

[14] Dasgupta S, Menezes ME, Das SK, Emdad L, Janjic A, Bhatia S, Mukhopadhyay ND, Shao C, Sarkar D, Fisher PB (2013). Novel role of MDA-9/syntenin in regulating urothelial cell proliferation by modulating EGFR signaling. Clinical cancer research.

[15] Kegelman TP, Das SK, Hu B, Bacolod MD, Fuller CE, Menezes ME, Emdad L, Dasgupta S, Baldwin AS, Bruce JN, Dent P, Pellecchia M, Sarkar D, Fisher PB (2014). MDA-9/syntenin is a key regulator of glioma pathogenesis. Neuro-oncology.

[16] Kim WY, Jang JY, Jeon YK, Chung DH, Kim YG, Kim CW (2014). Syntenin increases the invasiveness of small cell lung cancer cells by activating p38, AKT, focal adhesion kinase and SP1. Exp Mol Med.

[17] Liu X, Zhang X, Lv Y, Xiang J, Shi J (2014). Overexpression of syntenin enhances hepatoma cell proliferation and invasion: Potential roles in human hepatoma. Oncol Rep.

[18] Oyesanya RA, Bhatia S, Menezes ME, Dumur CI, Singh KP, Bae S, Troyer DA, Wells RB, Sauter ER, Sidransky D, Fisher PB, Semmes OJ, Dasgupta S (2014). MDA-9/Syntenin regulates differentiation and angiogenesis programs in head and neck squamous cell carcinoma. Oncoscience.

[19] Yang Y, Hong Q, Shi P, Liu Z, Luo J, Shao Z (2013). Elevated expression of syntenin in breast cancer is correlated with lymph node metastasis and poor patient survival. Breast cancer research.

[20] Qian XL, Li YQ, Yu B, Gu F, Liu FF, Li WD, Zhang XM, Fu L (2013). Syndecan binding protein (SDCBP) is overexpressed in estrogen receptor negative breast cancers, and is a potential promoter for tumor proliferation. PLoS One.

[21] Zardavas D, Baselga J, Piccart M (2013). Emerging targeted agents in metastatic breast cancer. Nat Rev Clin Oncol.

[22] Yamazaki D, Kurisu S, Takenawa T (2005). Regulation of cancer cell motility through actin reorganization. Cancer science.

[23] Debnath J, Muthuswamy SK, Brugge JS (2003). Morphogenesis and oncogenesis of MCF-10A mammary epithelial acini grown in three-dimensional basement membrane cultures. Methods.

[24] Nieman MT, Prudoff RS, Johnson KR, Wheelock MJ (1999). N-cadherin promotes motility in human breast cancer cells regardless of their E-cadherin expression. J Cell Biol.

[25] Suyama K, Shapiro I, Guttman M, Hazan RB (2002). A signaling pathway leading to metastasis is controlled by N-cadherin and the FGF receptor. Cancer cell.

[26] Zhang Z, Yang M, Chen R, Su W, Li P, Chen S, Chen Z, Chen A, Li S, Hu C (2014). IBP regulates epithelial-to-mesenchymal transition and the motility of breast cancer cells via Rac1, RhoA and Cdc42 signaling pathways. Oncogene.

[27] Sit ST, Manser E (2011). Rho GTPases and their role in organizing the actin cytoskeleton. Journal of cell science.

[28] Tapon N, Hall A (1997). Rho, Rac and Cdc42 GTPases regulate the organization of the actin cytoskeleton. Current opinion in cell biology.

[29] Bhowmick NA, Ghiassi M, Bakin A, Aakre M, Lundquist CA, Engel ME, Arteaga CL, Moses HL (2001). Transforming growth factor-beta1 mediates epithelial to mesenchymal transdifferentiation through a RhoA-dependent mechanism. Molecular biology of the cell.

[30] Zhang YE (2009). Non-Smad pathways in TGF-beta signaling. Cell research.

[31] Grootjans JJ, Zimmermann P, Reekmans G, Smets A, Degeest G, Durr J, David G (1997). Syntenin, a PDZ protein that binds syndecan cytoplasmic domains. Proc Natl Acad Sci U S A.

[32] Edlund S, Landstrom M, Heldin CH, Aspenstrom P (2002). Transforming growth factor-beta-induced mobilization of actin cytoskeleton requires signaling by small GTPases Cdc42 and RhoA. Molecular biology of the cell.

[33] Hwangbo C, Tae N, Lee S, Kim O, Park OK, Kim J, Kwon SH, Lee JH (2016). Syntenin regulates TGF-beta1-induced Smad activation and the epithelial-to-mesenchymal transition by inhibiting caveolin-mediated TGF-beta type I receptor internalization. Oncogene.

[34] Wang LK, Pan SH, Chang YL, Hung PF, Kao SH, Wang WL, Lin CW, Yang SC, Liang CH, Wu CT, Hsiao TH, Hong TM, Yang PC (2016). MDA-9/Syntenin-Slug transcriptional complex promote epithelial-mesenchymal transition and invasion/metastasis in lung adenocarcinoma. Oncotarget.

[35] Cheung SY, Boey YJ, Koh VC, Thike AA, Lim JC, Iqbal J, Tan PH (2015). Role of epithelial-mesenchymal transition markers in triple-negative breast cancer. Breast cancer research and treatment.

[36] Zeng Q, Li W, Lu D, Wu Z, Duan H, Luo Y, Feng J, Yang D, Fu L, Yan X (2012). CD146, an epithelial-mesenchymal transition inducer, is associated with triple-negative breast cancer. Proceedings of the National Academy of Sciences of the United States of America.

[37] Bhowmick NA, Zent R, Ghiassi M, McDonnell M, Moses HL (2001). Integrin beta 1 signaling is necessary for transforming growth factor-beta activation of p38MAPK and epithelial plasticity. J Biol Chem.

[38] Hwangbo C, Kim J, Lee JJ, Lee JH (2010). Activation of the integrin effector kinase focal adhesion kinase in cancer cells is regulated by crosstalk between protein kinase Calpha and the PDZ adapter protein mda-9/Syntenin. Cancer research.

[39] Lu P, Weaver VM, Werb Z (2012). The extracellular matrix: a dynamic niche in cancer progression. The Journal of cell biology.

[40] Pontier SM, Muller WJ (2009). Integrins in mammary-stem-cell biology and breast-cancer progression—a role in cancer stem cells?. Journal of cell science.

[41] Felding-Habermann B, O'Toole TE, Smith JW, Fransvea E, Ruggeri ZM, Ginsberg MH, Hughes PE, Pampori N, Shattil SJ, Saven A, Mueller BM (2001). Integrin activation controls metastasis in human breast cancer. Proc Natl Acad Sci U S A.

[42] Huck L, Pontier SM, Zuo DM, Muller WJ (2010). beta1-integrin is dispensable for the induction of ErbB2 mammary tumors but plays a critical role in the metastatic phase of tumor progression. Proc Natl Acad Sci U S A.

[43] White DE, Muller WJ (2007). Multifaceted roles of integrins in breast cancer metastasis. Journal of mammary gland biology and neoplasia.

[44] Lahlou H, Muller WJ (2011). beta1-integrins signaling and mammary tumor progression in transgenic mouse models: implications for human breast cancer. Breast Cancer Res.

[45] Hwangbo C, Park J, Lee JH (2011). mda-9/Syntenin protein positively regulates the activation of Akt protein by facilitating integrin-linked kinase adaptor function during adhesion to type I collagen. The Journal of biological chemistry.

[46] Holliday DL, Speirs V (2011). Choosing the right cell line for breast cancer research. Breast cancer research : BCR.

[47] Price JE (1996). Metastasis from human breast cancer cell lines. Breast cancer research and treatment.

[48] Chavez KJ, Garimella SV, Lipkowitz S (2010). Triple negative breast cancer cell lines: one tool in the search for better treatment of triple negative breast cancer. Breast disease.

[49] Das SK, Bhutia SK, Azab B, Kegelman TP, Peachy L, Santhekadur PK, Dasgupta S, Dash R, Dent P, Grant S, Emdad L, Pellecchia M, Sarkar D, Fisher PB (2013). MDA-9/syntenin and IGFBP-2 promote angiogenesis in human melanoma. Cancer research.

[50] Menezes ME, Mitra A, Shevde LA, Samant RS (2012). DNAJB6 governs a novel regulatory loop determining Wnt/beta-catenin signalling activity. Biochem J.

