# Supplementary material for: MDA-9/Syntenin (SDCBP) modulates small GTPases RhoA and Cdc42 via transforming growth factor β1 to enhance epithelial-mesenchymal transition in breast cancer
Source: Oncotarget. 2016 Nov 15;7(49):80175–89. doi: 10.18632/oncotarget.13373 (PMC5348312; doi:10.18632/oncotarget.13373)
Supplement: Supplementary file 1 [file oncotarget-07-80175-s001.pdf]

MDA-9/Syntenin (SDCBP) modulates small GTPases RhoA and Cdc42 *via* transforming growth factor β1 to enhance epithelial-mesenchymal transition in breast cancer

Supplementary Material

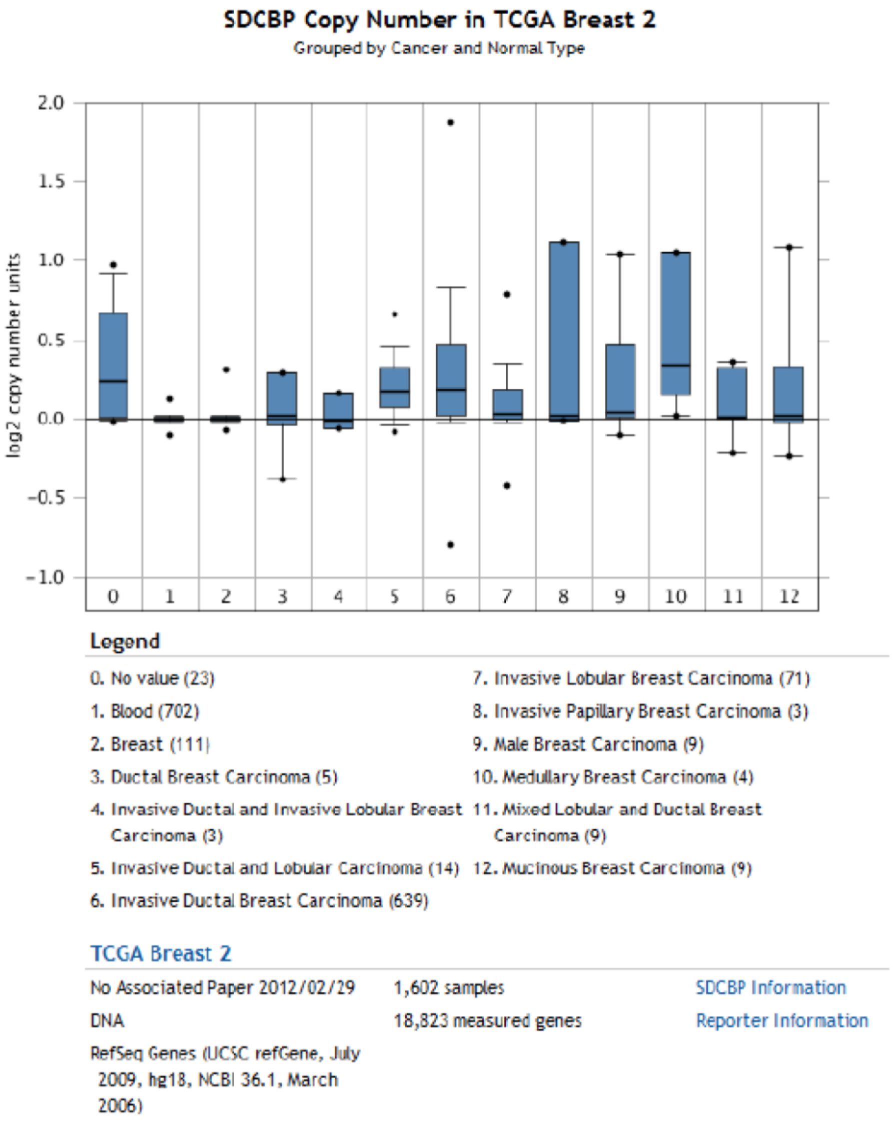

Supplementary Figure 1. DNA copy number of MDA-9 is elevated in human breast cancer patients. Histogram from TCGA database in Oncomine demonstrating MDA-9 copy number elevation in breast tumors compared to normal breast.

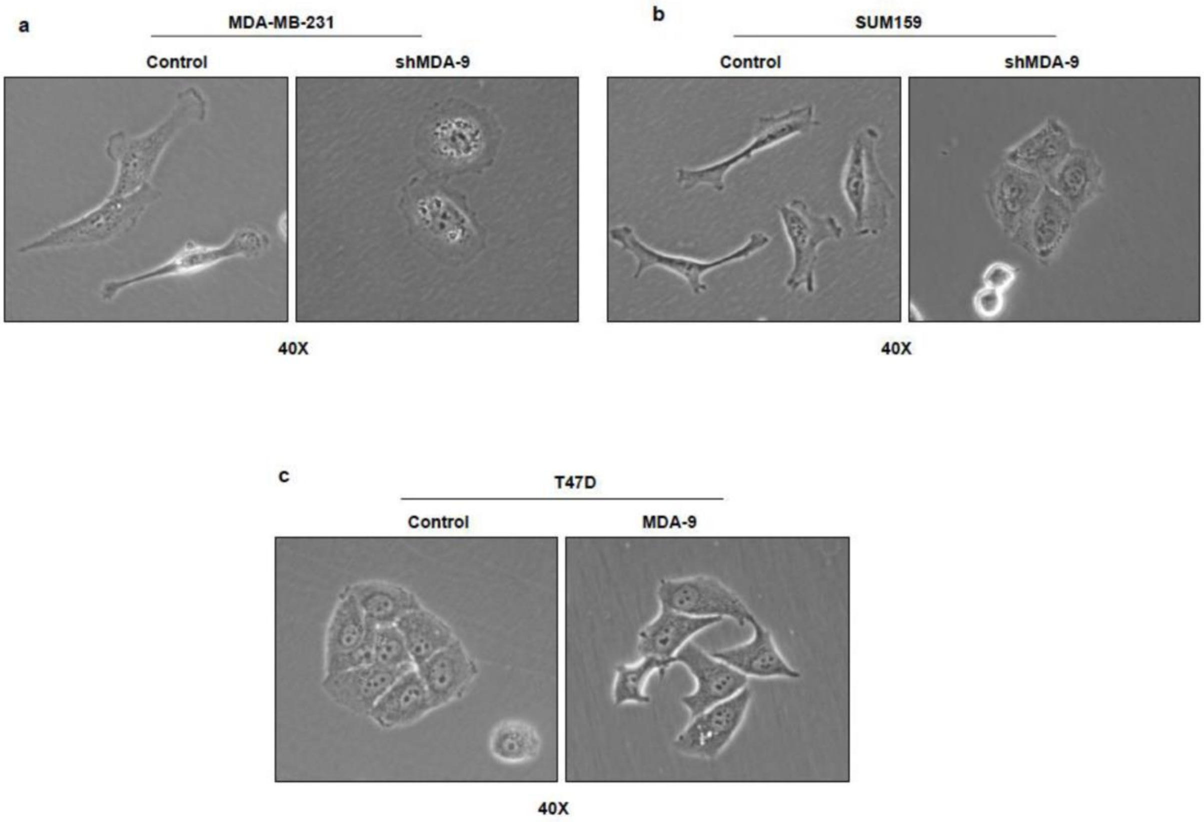

Supplementary Figure 2. Modulation of MDA-9 expression causes changes in cell shape. Representative images showing change in morphology in 2-dimensional culture on plastic plates following silencing MDA-9 expression in MDA-MB-231 and SUM159 and overexpressing MDA-9 in T47D cells.

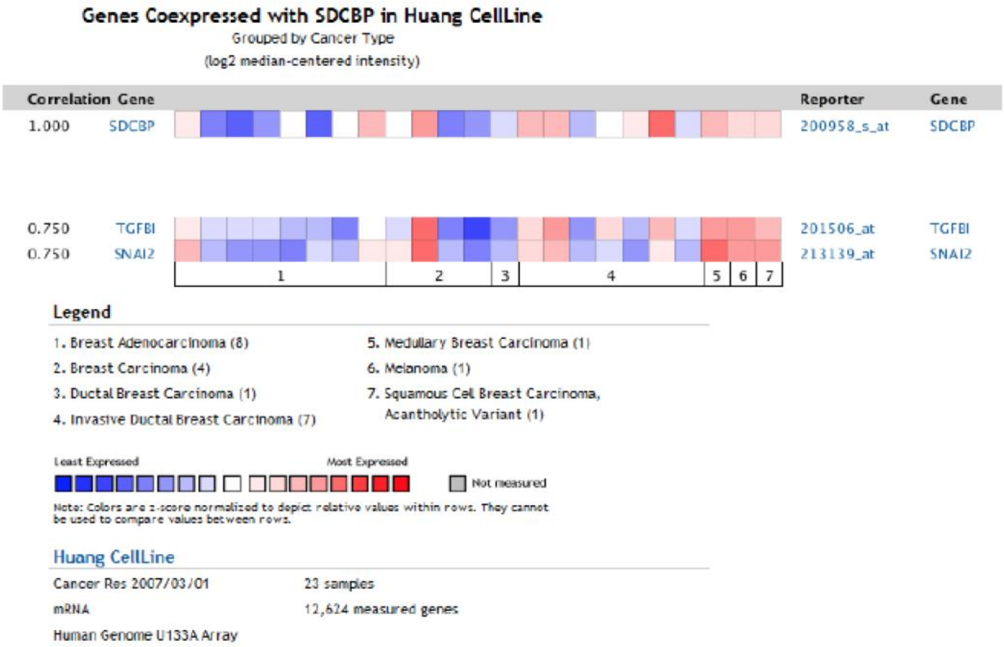

Supplementary Figure 3. MDA-9 and TGFβ1 are co-expressed in breast cancer patient samples. Correlation data from TCGA database in Oncomine demonstrating that MDA-9, TGFβ1 and SNAI2 (also known as Slug, a key EMT marker) genes are coexpressed in breast cancer patient samples.

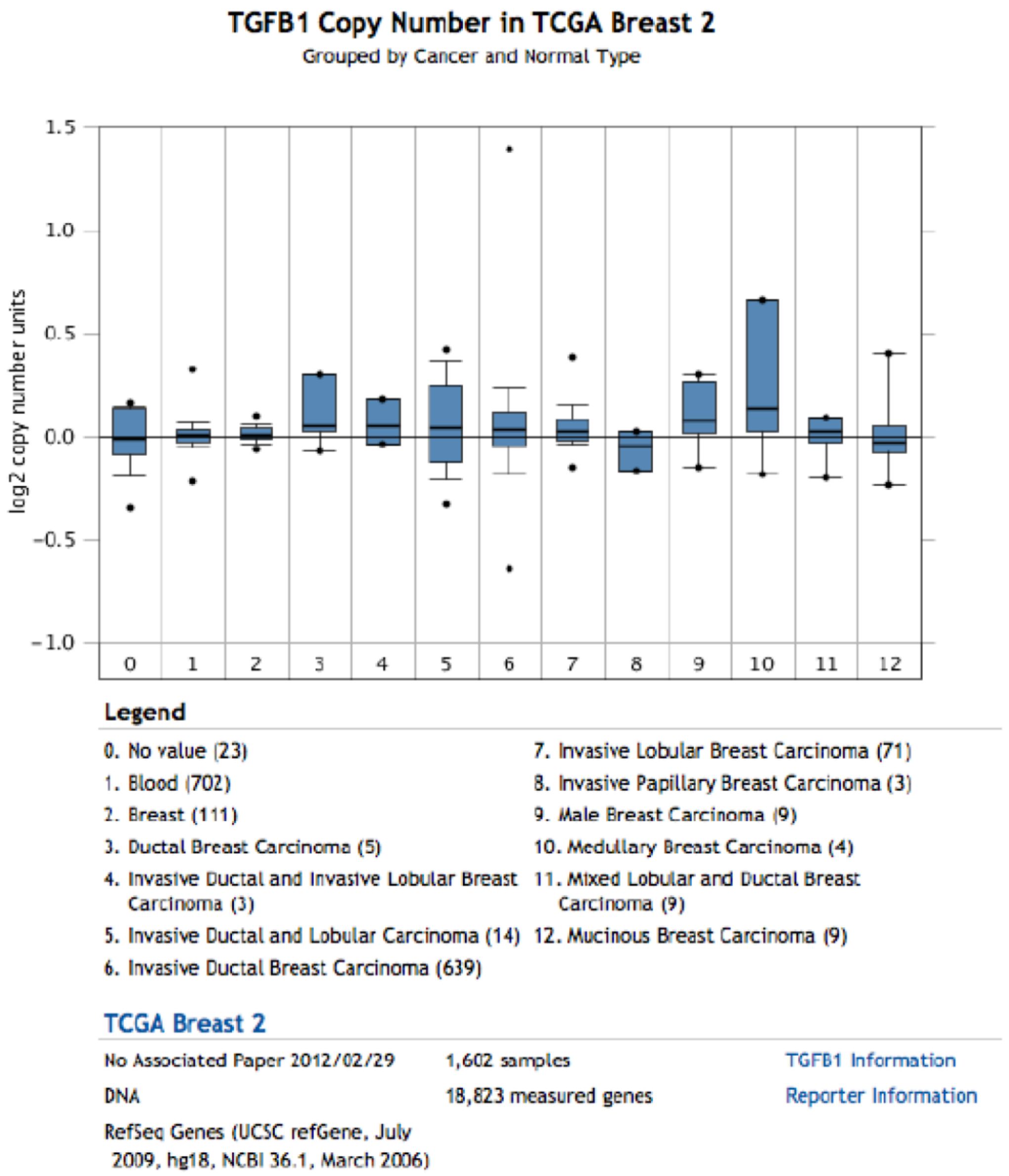

Supplementary Figure 4. DNA copy number of TGFβ1 is elevated in in human breast cancer patients. Histogram from TCGA database in Oncomine demonstrating TGFβ1 copy number elevation in breast tumors compared to normal breast in the same sample set assessed for MDA-9 expression in Supplementary Figure 1.

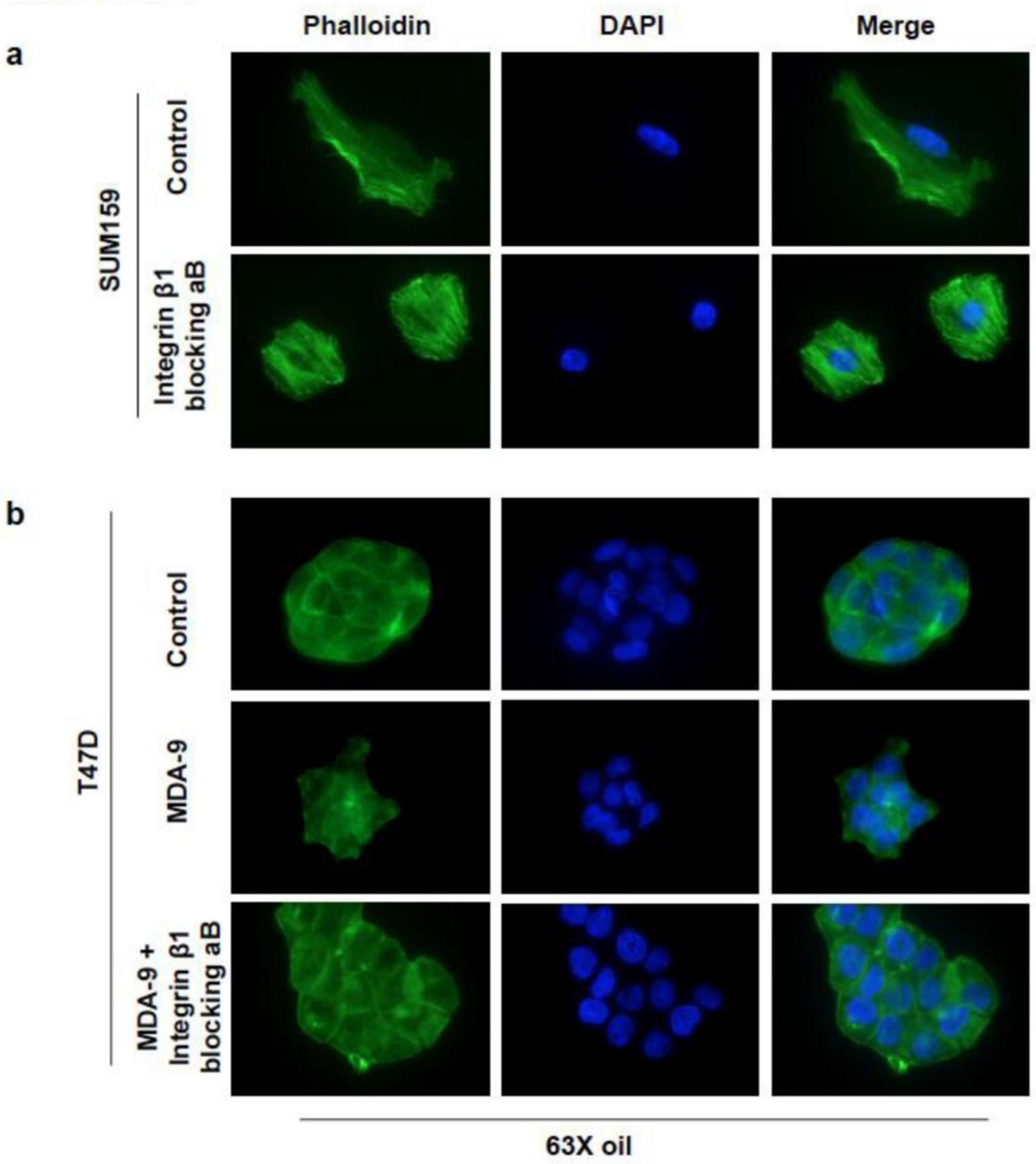

Supplementary Figure 5. Integrin β1 regulates cytoskeletal reorganization

(a) Representative images showing change in cytoskeletal reorganization following addition of Integrin β1 blocking antibody in SUM159 cells. (b) Representative images showing change in cytoskeletal reorganization following overexpression of MDA-9 that was restored upon addition of Integrin β1 blocking antibody.
